# Outer membrane vesicles of *Tannerella forsythia*: biogenesis, composition, and virulence

**DOI:** 10.1111/omi.12104

**Published:** 2015-06-16

**Authors:** V. Friedrich, C. Gruber, I. Nimeth, S. Pabinger, G. Sekot, G. Posch, F. Altmann, P. Messner, O. Andrukhov, C. Schäffer

**Affiliations:** ^1^ Department of NanoBiotechnology *NanoGlycobiology* unitUniversität für Bodenkultur WienViennaAustria; ^2^ Department of ChemistryUniversität für Bodenkultur WienViennaAustria; ^3^ AIT Austrian Institute of Technology, Health & Environment DepartmentMolecular DiagnosticsViennaAustria; ^4^ Division of Conservative Dentistry and PeriodontologyCompetence Centre of Periodontal ResearchUniversity Clinic of DentistryMedical University of ViennaViennaAustria; ^5^ Baxter AGViennaAustria; ^6^ GlycoVaxynSchlierenSwitzerland

**Keywords:** atomic force microscopy, electron microscopy, inflammatory response, outer membrane vesicles, periodontal pathogen, *Tannerella forsythia* strain ATCC 43037 *versus* strain 92A2

## Abstract

*Tannerella forsythia* is the only ‘red‐complex’ bacterium covered by an S‐layer, which has been shown to affect virulence. Here, outer membrane vesicles (OMVs) enriched with putative glycoproteins are described as a new addition to the virulence repertoire of *T. forsythia*. Investigations of this bacterium are hampered by its fastidious growth requirements and the recently discovered mismatch of the available genome sequence (92A2 = ATCC BAA‐2717) and the widely used *T. forsythia* strain (ATCC 43037). *T. forsythia* was grown anaerobically in serum‐free medium and biogenesis of OMVs was analyzed by electron and atomic force microscopy. This revealed OMVs with a mean diameter of ~100 nm budding off from the outer membrane while retaining the S‐layer. An LC‐ESI‐TOF/TOF proteomic analysis of OMVs from three independent biological replicates identified 175 proteins. Of these, 14 exhibited a C‐terminal outer membrane translocation signal that directs them to the cell/vesicle surface, 61 and 53 were localized to the outer membrane and periplasm, respectively, 22 were predicted to be extracellular, and 39 to originate from the cytoplasm. Eighty proteins contained the *Bacteroidales O*‐glycosylation motif, 18 of which were confirmed as glycoproteins. Release of pro‐inflammatory mediators from the human monocytic cell line U937 and periodontal ligament fibroblasts upon stimulation with OMVs followed a concentration‐dependent increase that was more pronounced in the presence of soluble CD14 in conditioned media. The inflammatory response was significantly higher than that caused by whole *T. forsythia* cells. Our study represents the first characterization of *T. forsythia* OMVs, their proteomic composition and immunogenic potential.

## Introduction

*Tannerella forsythia*,* Porphyromonas gingivalis* and *Treponema denticola* are Gram‐negative anaerobes that have been identified as major periodontal pathogens (Socransky *et al*., 1998). Together, they constitute the so‐called ‘red‐complex’ consortium that colonizes dental plaque biofilms (Holt & Ebersole, 2005) and is strongly associated with the clinical measures of periodontitis, a multifactorial, inflammatory disease of global importance (Darveau, 2010). According to a more recent model of pathogenesis, periodontitis is initiated by a synergistic and dysbiotic microbial community rather than by select periodontal pathogens, such as the ‘red complex’ (Hajishengallis & Lamont, 2012). In this polymicrobial synergy, different members or specific gene combinations within the community fulfill distinct roles that converge to shape and stabilize a disease‐provoking microbiota.

Apart from oral health issues, emerging evidence shows a relationship between periodontitis, cardiovascular disease, rheumatoid arthritis and other systemic chronic diseases (Cullinan *et al*., 2009; Koziel *et al*., 2014). For the development of new therapeutic strategies to combat periodontitis, a molecular understanding of the mechanisms governing bacterial virulence is required. Apart from distinct virulence factors that have been identified for the ‘red‐complex’ bacteria (O'Brien‐Simpson *et al*., 2004), outer membrane vesicles (OMVs), which are also known as integral parts of biofilm matrices (Flemming *et al*., 2007), are emerging as ‘bacterial warfare’ agents in the pathogenesis of periodontitis.

Generally, OMVs are natural secretion products of Gram‐negative bacteria, with an increasing number of pathogens being reported to release OMVs (Unal *et al*., 2011; Schertzer & Whiteley, 2013). They are small, spherical particles, usually 20–250 nm in diameter, and secreted throughout the bacterial life cycle and in a wide range of environmental conditions. Although the mechanism of OMV biogenesis is still poorly understood, studies so far point towards a highly regulated process that is most likely essential to the cell. Current models suggest that vesiculation occurs by budding off from the outer membrane (OM) at sites where lipoprotein links between the OM and the peptidoglycan are lost (Kulp & Kuehn, 2010). OMVs are characterized by selective enrichment or exclusion of specific cargo (Haurat *et al*., 2011); containing, apart from OM‐derived components such as lipopolysaccharide (LPS), phospholipids and OM proteins, also periplasmic constituents. OMVs are gaining increasing attention as a major mechanism by which pathogens attack and interact with host cells. As such, they have been shown to play roles in establishing colonization, carrying and transmitting virulence factors into host cells, and modulating host defense and response (Ellis & Kuehn, 2010).

Regarding the ‘red‐complex’ consortium, OMVs have so far only been described in detail for *P. gingivalis* (Veith *et al*., 2014), which is generally the best investigated ‘red‐complex’ bacterium. *P. gingivalis* OMVs are found in gingival tissues at diseased sites in chronic periodontitis but not at healthy sites (O'Brien‐Simpson *et al*., 2009) and are recognized as important virulence factors that are produced when *P. gingivalis* is part of a polymicrobial biofilm (Zhu *et al*., 2013). The *P. gingivalis* OMVs can invade host epithelial cells via an endocytic pathway (Furuta *et al*., 2009) and impair cellular functions by the gingipain‐exerted degradation of important receptor proteins (Veillard *et al*., 2012; Wilensky *et al*., 2015). In the context of the ‘red complex’, a synergistic effect of *P. gingivalis* OMVs has become obvious through their enhancement of *T. forsythia* attachment to epithelial cells (Inagaki *et al*., 2006). A recent study supports an interdependence between *P. gingivalis* virulence factors and *T. forsythia*, revealing that *P. gingivalis* gingipains influence the composition of polymicrobial biofilms (Bao *et al*., 2014).

For *T. forsythia*, the identification of new virulence factors (Sharma, 2010) is hampered by its fastidious growth requirements and the recently discovered mismatch of the available genome sequence (92A2=ATCC BAA‐2717) and the deposited *T. forsythia* strain (ATCC 43037) (Friedrich *et al*., 2015). So far, only a few putative virulence factors of *T. forsythia* have been identified. These include trypsin‐like (Amano *et al*., 2014) and PrtH (Saito *et al*., 1997) proteases, the sialidases SiaH (Horstman & Kuehn, 2000) and NanH (Thompson *et al*., 2009; Stafford *et al*., 2012), apoptosis‐inducing activity (Evans *et al*., 2012), α‐d‐glucosidase and *N*‐acetyl‐β‐glucosaminidase (Hughes *et al*., 2003), a hemagglutinin (Bomberger *et al*., 2009), methylglyoxal (Forsberg *et al*., 1981), a leucine‐rich repeat cell surface‐associated and secreted protein BspA (Sharma *et al*., 1998), the KLIKK proteases (Ksiazek *et al*., 2015b) such as karilysin (Karim *et al*., 2010; Koziel *et al*., 2010) and mirolase (Ksiazek *et al*., 2015a) as well as the S‐layer (Sabet *et al*., 2003). The latter belong to the major class of proteins carrying a C‐terminal OM translocation signal known as CTD (C‐terminal domain) (Lee *et al*., 2006; Sato *et al*., 2013; Narita *et al*., 2014; Tomek *et al*., 2014). Similarly to *P. gingivalis* and *Treponema denticola* (Dashper *et al*., 2011), *T. forsythia* secretes large amounts of CTD proteins by directing them to a type IX secretion system (T9SS), the presence of which has been demonstrated in *T. forsythia* only recently (Narita *et al*., 2014; Tomek *et al*., 2014). Some of these CTD proteins have been recognized as virulence factors (Veith *et al*., 2009b; Sharma, 2010). In this context it is interesting to note that both of the S‐layer proteins (TfsA and TfsB) as well as BspA are glycoproteins (Veith *et al*., 2009b). The S‐layer glycoproteins (TfsA–GP, TfsB‐GP) are intercalated on the bacterial cell surface to form a so far unique two‐dimensional crystalline monolayer (Sekot *et al*., 2012). For TfsA and TfsB it was shown experimentally that they are modified by the general protein *O*‐glycosylation system of *T. forsythia* by the addition of multiple copies of a complex oligosaccharide (Posch *et al*., 2011). A trisaccharide branch of this oligosaccharide, composed of two *N*‐acetylmannosaminuronic acid residues and one modified nonulosonic acid, was shown to modulate dendritic cell effector functions to suppress T‐helper 17 responses, thereby ensuring the persistence of the pathogen in the host (Settem *et al*., 2013). It is evident that several other *T. forsythia* proteins are targeted by the general *O‐*glycosylation system (Posch *et al*., 2011), with BspA likely to be among them.

To contribute to the understanding of virulence mechanisms of *T. forsythia*, this study was designed to shed light on the bacterium's OMVs and their putative role in shuttling virulence factors to reach distant cells in a concentrated, protected and targeted form. Here, (i) the biogenesis and morphology of *T. forsythia* OMVs were visualized by electron microscopy using ultrathin‐sectioned and negatively stained bacterial cells as well as isolated OMVs; the native situation of vesiculation was investigated by atomic force microscopy (AFM); (ii) a compositional analysis of the OMVs was performed; specifically, the content of S‐layer glycoproteins and LPS was determined; (iii) a shotgun proteomics approach was used to identify OMV proteins followed by categorizing them according to their predicted cellular location and their CTD OM translocation signal, with a focus on putative glycoproteins; and (iv) the release of proinflammatory mediators potentially relevant to the development of periodontitis [tumor necrosis factor‐α (TNF‐α), interleukin‐6 (IL‐6), IL‐8, monocyte chemoattractant protein 1 (MCP‐1)] from human macrophages and periodontal ligament fibroblasts was determined in comparison to whole *T. forsythia* cells. Our data support the virulent character of *T. forsythia* OMVs and indicate that they are enriched in putative glycoproteins, strengthening the importance of the interplay between glycobiology and virulence in this periodontal pathogen.

Considering that all published data on *T. forsythia* ATCC 43037 genes and proteins, so far, were assigned to the genome sequence of the wrong strain (92A2=ATCC BAA‐2717), it is another aim of this study to perform a profound comparison of our OMV proteome data, based on the correct, recently sequenced genome of *T. forsythia* ATCC 43037 (Friedrich *et al*., 2015), to the published OM proteome data of *T. forsythia* (Veith *et al*., 2009a). This should facilitate the identification of candidates for developing diagnostics and therapeutics against *T. forsythia* infection.

## Methods

### Bacterial strain and growth conditions

*Tannerella forsythia* wild‐type strain ATCC 43037 was purchased from ATCC (Manassas, VA) and grown anaerobically at 37°C for up to 7 days in 37 g l^−1^ of brain–heart infusion broth (Oxoid, Basingstoke, UK) supplemented with 10 g l^−1^ yeast extract (Oxoid), 1 g l^−1^ l‐cysteine (Sigma, Vienna, Austria), 2 μg ml^−1^ menadione (Sigma), 5 μg ml^−1^ hemin (Sigma) and 20 μg ml^−1^ *N*‐acetylmuramic acid (Carbosynth, Compton, UK). To avoid interference of serum proteins with immunological and proteomic analyses, no serum was added to the growth medium.

### Preparation of OMVs

The preparation of OMVs essentially followed a published protocol (Kadurugamuwa & Beveridge, 1995). Briefly, cells from a 200‐ml *T. forsythia* culture at an optical density at 600 nm (OD_600_) of ~1.0 were pelleted (20 min, 10,000 ***g***, 4°C) and the supernatant was sequentially filtered through a 0.45‐μm and a 0.22‐μm pore‐size PVDF membrane (Stericup‐GV, Millipore, Billerica, MA) to remove residual cell debris. Subsequently, the filtrate was subjected to ultracentrifugation (2 h, 100,000 ***g***, 4°C) to recover OMVs; the pellet was washed twice with sterile phosphate‐buffered saline (PBS), resuspended in 25 ml of PBS and ultracentrifuged again. For immunological studies, the pellet was finally resuspended in 2 ml of sterile PBS and stored in aliquots at −20°C. For proteomic analyses, the OMVs were resuspended in 10 μl Laemmli buffer (Laemmli, 1970) per mg of pellet.

### Microscopy

One milliliter of *T. forsythia* ATCC 43037 culture was centrifuged (5 min, 4500 ***g***, 4°C), washed twice with PBS and resuspended in 500 μl of PBS. Ten‐microliter aliquots of the concentrated sample were used for microscopic analyses. Transmission electron microscopy (TEM) of negatively stained and ultrathin‐sectioned preparations of *T. forsythia* cells as well as of isolated *T. forsythia* OMVs was performed on a Tecnai G2 20 Twin microscope (FEI, Eindhoven, the Netherlands) operating at 120 kV, as described previously (Messner *et al*., 1986; Sekot *et al*., 2012). For non‐invasive *in vitro* imaging by AFM using a Nanoscope III multimode AFM (Veeco Instruments Inc., Santa Barbara, CA), the sample was immobilized by mechanical trapping on a 0.8‐μm polycarbonate membrane (Millipore) (Oh *et al*., 2013) and imaged in contact mode with a DNP‐10 cantilever (Bruker, Vienna, Austria) with a nominal spring constant of 0.06 N m^−1^ and a nominal tip diameter of 20 nm. Scan line speed was 1 Hz.

### Biochemical methods

Sodium dodecyl sulfate–polyacrylamide gel electrophoresis (SDS–PAGE) of OMVs and crude *T. forsythia* cell extract was performed on 12% slab gels in a Mini Protean electrophoresis apparatus (Bio‐Rad, Vienna, Austria) (Laemmli, 1970). Proteins were stained with colloidal Coomassie Brilliant Blue R‐250 and glycans were visualized with periodic acid Schiff reagent. Western‐blotting of (glyco)proteins to a polyvinylidene difluoride membrane using anti‐TfsA and anti‐TfsB specific polyclonal antiserum was performed as described elsewhere (Steiner *et al*., 2007). Relative quantification of S‐layer proteins using OMV preparations equaling 5 μg, 0.5 μg and 0.05 μg of total protein was performed directly from the Western blots using an Odyssey scanner (LI‐COR, Bad Homburg, Germany) and integration of the intensity signal obtained from the S‐layer bands at 700 nm using the application software 3.0.

The protein content of the samples was determined with the 2‐D Quant kit (GE Healthcare, Vienna, Austria). To determine the LPS content of the OMV preparation used in the cell culture experiments, the amount of 3‐deoxy‐d‐*manno*‐oct‐2‐ulosonic acid (Kdo) was calculated (Warren, 1959).

### Mass spectrometry of OMVs

The OMV preparations from three independent biological replicates were separated in 10‐μl aliquots, each, by SDS–PAGE. Three zones were excised per lane and proteins were S‐alkylated with iodoacetamide and subsequently subjected to in‐gel digestion with modified trypsin (Promega, Mannheim, Germany). The peptide mixture was analyzed in the positive‐ion DDA mode (switching to MSMS mode for eluting peaks) using a Dionex Ultimate 3000 system (Thermo Scientific, Vienna, Austria) directly linked to a quadrupole time‐of‐flight mass spectrometer (MS) (Bruker maxis 4G ETD; Bruker) equipped with the captive spray source and nano‐booster set‐up. MS scans were recorded over a range of 150–2200 Da and the eight highest peaks were selected for fragmentation. Instrument calibration was performed using the ESI calibration mixture (Agilent, Santa Clara, CA). For separation of the peptides, a Thermo Acclaim PepMap300 RSLC C18 separation column (2‐μm particle size; 150 × 0.075 mm) was used with a Thermo Acclaim PepMap μ‐pre‐column. A gradient from 5% to 32% of solvent B (0.1% formic acid in acetonitrile) in solvent A (0.1% formic acid in HQ‐water) within 60 min was applied at a flow rate of 0.3 μl min^−1^, followed by a 10‐min gradient from 32% B to 70% B to promote elution of large peptides.

### Bioinformatic protein identification

The analysis files from MS were converted (using Data Analysis, Bruker) to XML files suitable for performing an MS/MS ion search with proteinscape (Mascot). The tandem mass spectrometry data were assigned to the protein sequences of *T. forsythia* ATCC 43037 as annotated by the NCBI Prokaryotic Genome Annotation Pipeline. The Mascot search results of each gel lane were merged. Positive hits were accepted with at least two peptides per protein and a Mascot score of at least 30. In Table 1, only proteins that could be identified in all three replicate samples are listed. The total Mascot score was averaged across all samples and gel lanes.

**Table 1:** Identification and localization of outer membrane vesicle (OMV) proteins

Locus tag	Description	MW (kDa)	Glyco‐sites^a^	Predicted location^b^	Ortholog in strain 92A2	Mascot score^c^	Previously reported in OM proteome^d^
*C‐terminal‐domain‐containing proteins*							
Tanf_03375	surface layer protein TfsB	150.4	16*	P	TF2663	2735	+
Tanf_04820	surface antigen BspA	123.3	1*	P	TF1843	2330	+
Tanf_03370	surface layer protein TfsA	133.1	11*	OM	TF2661	2052	+
Tanf_06020	possible hemagglutinin/hemolysin	131.2	6*	E	TF2116	1381	+
Tanf_03675	alkyl hydroperoxide reductase	39.1	1	C	TF2730	730	
Tanf_10675	hypothetical protein	41.9	4	P	TF1273	659	
Tanf_05655	hypothetical protein	58.2	0	E	TF1552	512	
Tanf_12435	immunoreactive antigen PG93	84.8	0	E	TF1952	393	
Tanf_05510	hypothetical protein	41.1	3	OM	TF1514	358	
Tanf_02330	hypothetical protein	200.0	13	E	TF2320	253	+
Tanf_10605	hypothetical protein	34.0	0	C	TF2339^e^	232	+
Tanf_03310	bacterial group 2 Ig‐like protein	41.0	9	P	TF2645	174	
Tanf_12195	hypothetical protein	38.8	0	OM	TF1896	103	
Tanf_00535	hypothetical protein	212.7	16	E	TF3163	88	+
*Hydrolytic enzymes*							
Tanf_04930	peptidase, S41 family	54.1	3	OM	TF3024	842	+
Tanf_11420	periplasmic serine protease HtrA	54.2	0	OM	TF1450	762	
Tanf_00250	peptidase PepD, family C69	61.8	1	P	TF0298	513	
Tanf_04010	probable secreted glycosyl hydrolase	32.6	3*	P	TF2804	439	+
Tanf_00095	alkaline phosphatase	62.7	1	P	TF0338	437	
Tanf_03780	zinc protease	107.5	2*	C	TF2753	374	
Tanf_02020	endonuclease	31.5	0	C	TF1471	307	
Tanf_10220	peptidase family M49	78.4	1	C	TF2531	291	+
Tanf_04515	β‐*N*‐acetylglucosaminidase HexA	86.7	2	C	TF2925	290	+
Tanf_09025	peptidase, S41 family	48.8	2	OM	TF1243	282	
Tanf_08225	metalloendopeptidase PepO	77.3	3	P	TF1033	267	+
Tanf_10855	lysophospholipase	28.8	2	P	TF1313	262	
Tanf_07615	putative alkaline protease AprF	52.1	0	C	TF0773	261	+
Tanf_05640	xylanase	32.3	0	C	TF1549	236	
Tanf_13640	Icc family phosphohydrolase	36.2	0	C	TF0048	221	
Tanf_11165	peptidase S10	81.4	2	C	TF1398	183	
Tanf_07795	glycosyl hydrolase	48.5	1	C	TF0813	166	+
Tanf_13700	sialidase (neuraminidase) NanH	59.7	2	OM	TF0035	132	
Tanf_08515	peptidase, S41 family	39.1	2	C	TF1755	122	+
Tanf_13550	aminopeptidase C1‐like family	45.1	0	E	TF0078	109	
Tanf_06530	miropsin‐2	80.2	0	OM	TF0357^e^	104	
Tanf_02565	peptidase S41	121.8	0	P	TF0959	97	+
Tanf_06225	forsilysin	91.3	2	E	TF2162	94	
Tanf_02010	β‐galactosidase	128.4	1	E	TF1468	78	
Tanf_02130	exo‐α‐sialidase SiaHI	51.0	1	P	TF2207	41	+
*Type IX secretion system proteins*							
Tanf_04220	integral OM protein LptO	43.6	0	OM	TF2852	1025	+
Tanf_02580	putative C‐terminal signal peptidase	127.6	3	OM	TF0955	750	+
Tanf_12465	PorQ protein	37.2	0	OM	TF1959	184	+
Tanf_10520	PorT protein	27.1	0	OM	TF0188	102	
*TonB‐associated outer membrane proteins*							
Tanf_13710	neuraminate OM permease NanO	121.5	0	OM	TF0033	3000	
Tanf_08335	OM protein Omp121	113.7	0	OM	TF1057	2063	+
Tanf_13705	neuraminate uptake protein NanU	59.5	3	OM	TF0034	1880	
Tanf_09365	SusC/RagA family TonB‐linked OM protein	117.9	0	OM	TF2193	1328	+
Tanf_09370	SusD family protein	76.2	0	OM	TF2192	1313	+
Tanf_09520	TonB‐dependent receptor HmuY	24.5	0	P	TF3077	1088	
Tanf_00785	TonB‐linked OM protein	118.0	0	OM	TF3104	1009	+
Tanf_02485	SusC/RagA family TonB‐linked OM protein	117.7	1	OM	TF0976	939	+
Tanf_08320	OM protein Omp121	117.4	0	OM	TF1053	890	+
Tanf_00180	SusC/RagA family TonB‐linked OM protein	110.3	0	OM	TF0313	866	+
Tanf_13480	SusD family protein	58.7	0	OM	TF0092	858	+
Tanf_13475	OM protein SusC	112.3	0	OM	TF0093	835	+
Tanf_03665	SusC/RagA family TonB‐linked OM protein	133.1	1	OM	TF2728	807	+
Tanf_06285	SusC/RagA family TonB‐linked OM protein	110.9	0	OM	TF2348	759	+
Tanf_00790	SusD family protein	45.3	0	C	TF3103	678	+
Tanf_00185	SusD family protein	64.5	0	P	TF0312	595	+
Tanf_00330	SusD family protein	61.6	0	P	TF0277	520	+
Tanf_06290	SusD/RagB family OM lipoprotein	61.6	0	P	TF2349	511	+
Tanf_09940	OM protein Omp121	116.3	0	OM	TF2417	468	+
Tanf_09960	SusC/RagA family TonB‐linked OM protein	118.6	0	OM	TF2412	431	+
Tanf_03670	SusD family protein	54.9	0	C	TF2729	419	+
Tanf_02480	SusD family protein	63.5	0	C	TF0977	408	+
Tanf_12670	SusC/RagA family TonB‐linked OM protein	129.2	0	OM	TF0588	381	+
Tanf_13485	OM protein SusE	41.8	1*	E	TF0091	376	+
Tanf_00335	SusC/RagA family TonB‐linked OM protein	111.7	0	OM	TF0275	357	+
Tanf_05475	SusD family protein	60.5	0	OM	TF1505	324	+
Tanf_11255	TonB‐dependent receptor FrrG, OM protein	121.4	0	OM	TF1415	280	+
Tanf_01380	SusC/RagA family TonB‐linked OM protein	114.0	0	OM	TF2032	276	+
Tanf_07645	SusD family protein	54.2	0	P	TF0779	256	+
Tanf_00170	TonB‐dependent receptor	87.3	1	OM	TF0318	252	+
Tanf_07640	SusC/RagA family TonB‐linked OM protein	114.1	0	OM	TF0778	212	+
Tanf_06060	probable TonB‐linked OM protein	68.4	2	OM	TF2124	208	+
Tanf_13670	TonB‐dependent siderophore receptor	88.2	4	OM	TF0041	199	+
Tanf_00240	TonB‐dependent receptor	97.3	2	OM	TF0301	191	+
Tanf_01385	SusD/RagB family OM lipoprotein	62.2	1	E	TF2031	176	+
Tanf_12665	SusD family protein	58.1	0	P	TF0587	167	+
Tanf_03660	SusD family protein	65.3	0	OM	TF2727	95	+
Tanf_11260	SusD family protein	65.3	0	P	TF1416	79	+
Tanf_07035	SusD family protein	67.3	1	OM	TF0483	60	+
Tanf_07030	SusC/RagA family TonB‐linked OM protein	119.6	0	OM	TF0482	46	+
*Outer membrane proteins*							
Tanf_09620	OM protein transport protein	46.9	2	OM	TF0706	534	+
Tanf_01080	Oar‐like OM protein, OmpA family	121.1	0	OM	TF2096	321	+
Tanf_09805	OM protein assembly complex	102.6	3	OM	TF2450	227	+
Tanf_10935	OM protein 40, OmpA‐like	44.4	1	P	TF1331	180	+
Tanf_01635	copper resistance lipoprotein NlpE	15.9	0	C	TF1158	126	+
Tanf_09795	cationic OM protein OmpH	19.0	1*	P	TF2452	117	
Tanf_00475	possible immunogenic lipoprotein	23.9	0	P	TF0015	103	+
Tanf_06695	OM assembly lipoprotein YfiO	31.9	0	OM	TF0403	71	
*Other proteins*							
Tanf_11435	sugar phosphate isomerase/epimerase	33.8	0	C	TF1454	967	
Tanf_04005	oxidoreductase domain protein	54.8	1	P	TF2803	963	+
Tanf_10630	PDZ domain signal protein	54.9	1	OM	TF1262	518	
Tanf_07655	protease inhibitor miropin	45.7	0	C	TF0781	490	
Tanf_13170	putative peptidyl‐prolyl isomerase	61.7	4*	C	TF2506	426	
Tanf_07420	sporulation and cell division repeat protein	17.7	0	P	TF1733	406	+
Tanf_06265	glutathione peroxidase	22.6	0	P	TF2342	357	
Tanf_04530	superoxide dismutase SodF	22.1	0	C	TF2927	338	
Tanf_02155	hypothetical protein	21.6	0	P	TF2214	297	+
Tanf_05520	alkyl hydroperoxide reductase	22.5	0	P	TF1518	284	
Tanf_13665	anaerobic cobalt chelatase CbiK	35.3	0	C	TF0042	245	
Tanf_00220	peptidyl‐prolyl *cis‐trans* isomerase SlyD	26.5	2	P	TF0305	210	+
Tanf_13180	peptidyl‐prolyl *cis‐trans* isomerase SurA	52.5	3	C	TF2504	203	
Tanf_13630	flavodoxin	21.1	0	P	TF0054	176	
Tanf_08680	aldose 1‐epimerase GalM	41.3	3	E	TF1798	148	
Tanf_00150	possible YngK protein	60.4	1	C	TF0322	89	+
Tanf_07430	oxidoreductase domain protein	47.2	0	P	TF1735	86	
Tanf_00210	hydroxypyruvate isomerase	33.0	0	C	TF0307	74	
*Hypothetical proteins*							
Tanf_13655	bacterial group 2 Ig‐like protein	47.6	0	P	TF0044	1931	+
Tanf_02425	hypothetical protein	159.9	13*	E	TF2339	1515	+
Tanf_03910	hypothetical protein	23.2	0	P	none	1315	
Tanf_12375	tetratricopeptide repeat protein	62.0	0	P	TF1940	1285	+
Tanf_08330	tetratricopeptide repeat protein	71.2	2*	P	TF1056	1220	+
Tanf_09615	tetratricopeptide repeat protein	51.2	1	P	TF0704	1072	
Tanf_04855	OM protein β‐barrel domain protein	22.6	0	OM	TF3007	951	+
Tanf_11390	hypothetical, putative hemin receptor	60.4	0	OM	TF1444	879	+
Tanf_13105	Cna protein B‐type domain protein	48.8	0	E	TF0683	864	+
Tanf_07305	tetratricopeptide repeat protein	46.5	1*	P	TF0548	777	
Tanf_10775	putative internalin A	39.3	0	P	TF1294	773	
Tanf_02630	putative surface protein	40.4	2	E	TF0945	704	+
Tanf_13015	hypothetical protein	29.5	1	P	TF0661	694	+
Tanf_08325	hypothetical protein	32.6	2	P	TF1055	618	+
Tanf_08965	hypothetical protein	171.4	11*	E	TF1259	453	+
Tanf_10985	possible lipoprotein	54.5	6*	OM	TF1342	452	+
Tanf_10190	hypothetical protein	27.8	2	C	TF2537	429	
Tanf_03150	hypothetical protein	44.6	0	P	TF2606	407	+
Tanf_06055	tetratricopeptide repeat protein	114.5	2	OM	TF2123	394	+
Tanf_08315	hypothetical protein	67.5	2	E	TF1052	391	+
Tanf_10595	hypothetical protein	54.0	2	C	TF0358	390	
Tanf_03025	hypothetical protein	17.7	0	C	TF1714	390	
Tanf_06535	hypothetical protein	23.0	0	E	TF0358	376	
Tanf_09820	putative lipoprotein	51.2	5	OM	TF2447	366	+
Tanf_09445	hypothetical protein	16.0	0	P	TF0365	362	+
Tanf_06545	hypothetical protein	16.2	0	P	TF0365	350	+
Tanf_07845	hypothetical protein	21.7	0	P	TF0827	324	
Tanf_09945	hypothetical protein	58.1	0	P	TF2416	321	+
Tanf_13280	hypothetical protein	63.0	0	OM	TF2485	310	
Tanf_12860	hypothetical protein	54.8	0	OM	TF0627	299	
Tanf_05505	hypothetical protein	54.1	0	OM	none	296	
Tanf_00990	hypothetical protein	61.5	2*	E	TF2592^e^	293	+
Tanf_02915	hypothetical protein	56.1	2	C	TF1689	273	
Tanf_12975	hypothetical protein	79.0	5	OM	TF0652	259	+
Tanf_05585	hypothetical protein	18.2	0	P	TF1534	256	+
Tanf_12970	hypothetical protein	36.2	0	OM	TF0651	255	
Tanf_11375	hypothetical protein	23.5	0	OM	TF1441	244	+
Tanf_01450	hypothetical protein	17.7	0	P	TF2016	228	+
Tanf_05560	hypothetical protein	13.5	0	P	TF1527	210	
Tanf_01050	hypothetical protein	20.1	0	C	TF0860	205	
Tanf_00065	hypothetical protein	65.2	2*	E	TF1741	200	+
Tanf_12120	hypothetical protein	26.0	0	OM	TF1857	199	
Tanf_03695	hypothetical protein	23.0	0	OM	TF2734	178	+
Tanf_12265	hypothetical protein	25.1	0	C	TF1911	176	
Tanf_12880	bacterial group 2 Ig‐like protein	27.4	0	P	TF0631	175	
Tanf_09950	hypothetical protein	25.3	1	OM	TF2415	168	+
Tanf_03830	hypothetical protein	29.2	2	C	TF2764	161	
Tanf_07425	hypothetical protein	126.6	0	P	TF1734	153	
Tanf_00445	hypothetical protein	15.6	0	C	TF0342	148	
Tanf_08510	hypothetical protein	31.2	0	E	TF1754	142	
Tanf_07010	hypothetical protein	39.8	0	C	TF0479	139	
Tanf_06555	hypothetical protein	13.3	0	C	TF0368	129	+
Tanf_11855	hypothetical protein	67.4	3*	E	TF2592	124	+
Tanf_13010	hypothetical protein	49.4	1	OM	TF0660	123	
Tanf_10145	WD40‐like protein	54.4	3	C	TF2369	116	
Tanf_00660	hypothetical protein	44.7	3	C	TF3133	114	
Tanf_07095	hypothetical protein	52.2	0	P	TF0495	111	
Tanf_10060	hypothetical protein	55.8	1	P	TF2390	107	
Tanf_08090	hypothetical protein	23.5	0	P	TF1003	92	
Tanf_03090	hypothetical protein	33.1	6	C	TF2594	88	
Tanf_00865	hypothetical protein	32.6	2	P	TF3086^e^	87	
Tanf_05230	hypothetical protein	21.8	0	C	TF0137	83	
Tanf_07265	bacterial group 2 Ig‐like protein	38.4	1	E	TF0540	82	
Tanf_09955	hypothetical protein	26.1	2*	P	TF2414	81	+
Tanf_03095	hypothetical protein	59.1	1	OM	TF2595	77	+
Tanf_11080	hypothetical protein	39.7	2	C	TF1392	67	

a Number of occurrences of the phylum‐wide glycosylation motif. Asterisks denote proteins that have been reported to be glycosylated.

b CELLO prediction of subcellular localization: Periplasmic (P), Outer membrane (OM), Extracellular (E), Cytoplasmic (C).

c Total Mascot score averaged across the replicate samples.

d Veith *et al*. (2009b).

e More than one possible ortholog found, only best hit shown.

To find orthologs of the ATCC 43037 strain within the widely used 92A2 (ATCC BAA‐2717) genome, pBLAST was used to search all ‘Tanf’ ATCC 43037 protein sequences against both the ‘TF’ Oralgen (http://www.oralgen.org) and the ‘BFO’ NCBI (http://www.oralgen.org) annotated proteomes. In addition to an *E*‐value cut‐off of 1 × 10^−5^, the parameters ‘best_hit_score_edge 0.1’ and ‘best_hit_overhang 0.1’ were applied to select for only the best hits (BLAST Command Line Applications User Manual http://www.ncbi.nlm.nih.gov/books/NBK21097).

Function prediction was performed using PANNZER (bitscore threshold 50, identity threshold 50%, query and target coverage 0.6) (Koskinen *et al*., 2015). ScanPROSITE (http://prosite.expasy.org/scanprosite) was used to identify potential glycosylation sites by searching the *T. forsythia* proteome for the phylum‐wide glycosylation motif D(S,T)(A,L,V,I,M,T) (Coyne *et al*., 2013).

Proteins detected in the *T. forsythia* OMVs were classified according to their subcellular localization, which was established using the bacterial localization prediction software CELLO (http://meme-suite.org/tools/glam2scan) (Yu *et al*., 2004).

The prediction of CTD proteins was conducted by running a BLASTP analysis of known *T. forsythia* CTD proteins in strain 92A2 (Veith *et al*., 2009b) against the new genome of strain ATCC 43037. Furthermore, using previously described CTD sequences, a GLAM2 motif was created and subsequently used in GLAM2Scan (http://meme-suite.org/tools/glam2scan) to confirm positive hits.

### Cell lines

The U937 human monocytic cell line was purchased from ATCC and primary human periodontal ligament fibroblasts (hPdLFs) isolated from 16‐year old male donors were purchased from Lonza (Basel, Switzerland). Both cell lines were cultured in RPMI‐1640 medium (Invitrogen, Vienna, Austria) and Dulbecco's modified Eagle's medium (Invitrogen), respectively, supplemented with 10% of fetal calf serum (FCS), 100 U ml^−1^ penicillin, and 100 μg ml^−1^ streptomycin at 37°C in a humidified atmosphere containing 5% CO_2_. For differentiation into adherent macrophages, U937 cells were seeded (10^6^ cells ml^−1^) and treated with phorbol 12‐myristate 13‐acetate (Sigma) as described elsewhere (Sekot *et al*., 2011).

### Stimulation of macrophages and human gingival fibroblasts with OMVs

Adherent U937 macrophages were seeded in a 24‐well plate at a density of 2 × 10^5^ cells per well containing 0.5 ml of RPMI‐1640 medium, and hPdLFs were seeded at a density of 5 × 10^4^ cells per well containing 0.5 ml of Dulbecco's modified Eagle's medium; either medium was additionally supplemented with 10% FCS. After 24 h, the media were replaced with low‐serum media (1% FCS) containing different amounts of OMVs (corresponding to 0.1 μg, 1.0 μg and 10 μg protein per ml).

In a parallel set of experiments, human soluble CD14 (sCD14, 0.25 μg ml^−1^; Sigma, St Louis, USA) was added during stimulation. Cells stimulated with viable wild‐type *T. forsythia* (10^7^ cells ml^−1^) (Bodet *et al*., 2006) were used as a positive control. Each experimental group included three wells. After stimulation of U937 for 3 h and of hPdLFs for 24 h, the cellular mRNA expression levels of TNF‐α and IL‐8 in macrophages, and of IL‐6, IL‐8 and MCP‐1 in hPdLFs, as well as the content of the corresponding proteins in the conditioned medium were determined. Experiments were repeated at least three times.

### Viability test of mammalian cells

U937 macrophages and hPdLFs were treated with OMVs as described under ‘Stimulation of macrophages and human gingival fibroblasts with OMVs’ above. Additionally, PBS was used as a control. Each experimental group included eight wells. After stimulation, 10 μl of 3‐(4,5‐dimethylthiazol‐2‐yl)‐2,5‐diphenyltetrazolium bromide (MTT) dye (5 mg ml^−1^ in PBS) was added and culture plates were incubated at 37°C for 4 h. Subsequently, the medium was discarded, 100 μl of dimethylsulfoxide was added, and the OD_550_ was measured on a Spectramax Plus microplate reader (Molecular Devices, Sunnyvale, CA).

### Quantitative polymerase chain reaction

The mRNA expression levels of TNF‐α, IL‐6, IL‐8, and MCP‐1 were determined by quantitative polymerase chain reaction (qPCR), with glyceraldehyde 3‐phosphate‐dehydrogenase (GAPDH) and β‐actin serving as internal references. Isolation of mRNA and transcription into cDNA was performed using the TaqMan Gene Expression Cells‐to‐CT kit (Ambion/Applied Biosystems, Foster City, CA), which provides good accuracy and superior sensitivity of gene expression analysis (Van Peer *et al*., 2012). The qPCR was performed on an ABI StepOnePlus device (Life Technologies, Carlsbad, CA) in paired reactions using the TaqMan gene expression assays with the following ID numbers (Life Technologies): TNF‐α, Hs99999043_m1; IL‐6, Hs00985639_m1; IL‐8, Hs00174103_m1; MCP‐1, Hs00234140_m1; GAPDH, Hs99999905_m1; β‐actin, Hs99999903_m1. The qPCR were performed in triplicate as described previously (Sekot *et al*., 2011). The point at which the PCR product was first detected above a fixed threshold (cycle threshold, C_t_), was determined for each sample. Changes in the expression of target genes were calculated using the 2^−ΔΔCt^ method, using an untreated sample as control (Sekot *et al*., 2011).

### Determination of cytokines

The levels of TNF‐α, IL‐6, IL‐8, and MCP‐1 in the conditioned media were determined by enzyme‐linked immunosorbent assay using Ready‐SET‐Go kits (eBioscience, San Diego, CA). For measurement of TNF‐α and IL‐8 production by U937 macrophages, samples were diluted at a ratio of 1 : 10 and 1 : 100, respectively; for measurements of IL‐6 and MCP‐1 production by hPdLFs samples were used undiluted and for measurement of IL‐8 production samples were diluted at a the ratio of 1 : 5. The detection limit for all cytokines was 2 pg ml^−1^.

### Statistical analysis

The statistical difference between groups was determined by analysis of variance, while paired comparisons were performed using Tukey's post‐hoc test. All statistical analyses were performed using the statistics program SPSS 20.0. Data are expressed as mean ± SD. Differences were considered to be statistically significant at *P* < 0.05.

## Results and Discussion

### Microscopy of *T. forsythia* OMVs

**Figure 1:**
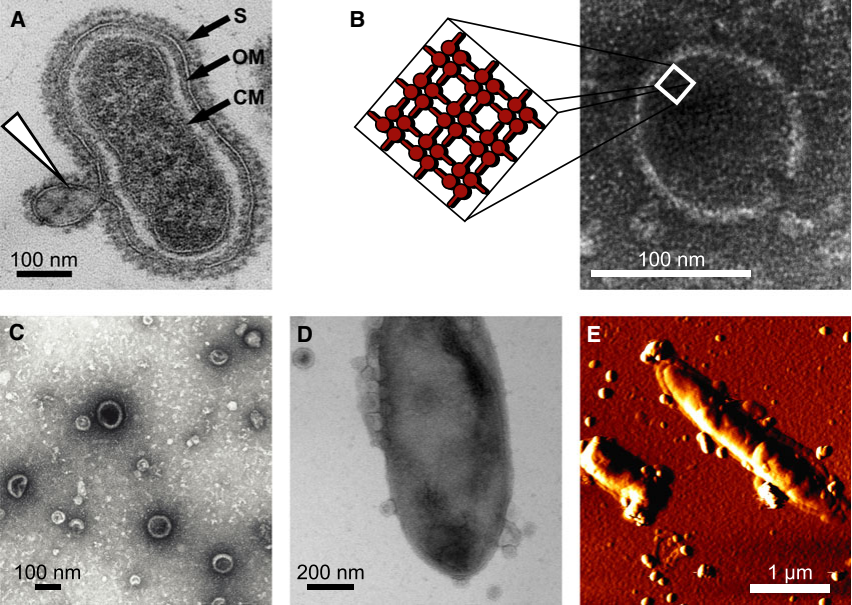
Transmission electron microscope images (A–D) and atomic force microscopy image (E) of *Tannerella forsythia* ATCC 43037 wild‐type cells and outer membrane vesicles (OMVs). (A) Ultrathin‐sectioned preparation of a cell showing the biogenesis of an OMV (white triangle) from the outer membrane and coverage of the nascent OMV with the S‐layer. (B) Negatively‐stained preparation of isolated *T. forsythia* OMVs showing the square S‐layer lattice (inset) and (C) the size distribution of the OMVs. (D) Negatively stained and (E) untreated, physically trapped *T. forsythia* cell showing nascent OMVs in the immediate surroundings of the cells. S, S‐layer; OM, outer membrane; CM, cytoplasmic membrane.

The TEM of ultrathin‐sectioned *T. forsythia* cells grown in serum‐free brain–heart infusion broth with a mean generation time of 16 h^−1^ revealed that OMV formation occurred by budding off from the OM, with the S‐layer completely covering the nascent vesicles (Fig. 1A). The S‐layer of OMVs exhibits a thickness of 22 nm, which conforms to the value measured from whole *T. forsythia* cells (Sekot *et al*., 2012). The presence of an intact S‐layer was supported by analysis of a negatively stained preparation of isolated OMVs, on which the square S‐layer lattice symmetry typical of *T. forsythia* cells was visible (Fig. 1B). The analyses of OMVs from intact OMV‐secreting cells as well as purified preparations revealed an almost uniform diameter of ~100 nm (Fig. 1C,D). These findings are supported by non‐invasive AFM *in vitro* imaging of OMV‐secreting *T. forsythia* cells, where cells neither received any chemical treatment nor were subjected to a drying process, which may trigger undesirable biological side effects (Oh *et al*., 2013). There, OMVs could be clearly visualized in the immediate surroundings as well as lying on top of the imaged *T. forsythia* cell (Fig. 1E).

### Biochemical analyses of *T. forsythia* OMVs

The *T. forsythia* OMV preparation used in the course of this study revealed an overall protein concentration of 2 μg ml^−1^, based on the 2‐D Quant kit, and a Kdo content of 16 ng ml^−1^.

**Figure 2:**
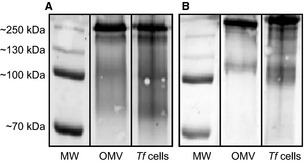
Western blot analysis for detection of the S‐layer glycoproteins TfsA‐GP and TfsB‐GP in the outer membrane vesicle preparation (OMV) and, for comparison, on whole *Tannerella forsythia* ATCC 43037 cells (Tf cells), using anti‐TfsA (A) and anti‐TfsB (B) antibodies. The S‐layer proteins were subsequently quantified using the LI‐COR Odyssey scanner. MW, molecular weight marker PageRuler Plus Prestained Protein Ladder (Thermo Scientific, Vienna, Austria).

The presence of an S‐layer as the outermost layer of the *T. forsythia* OMVs (compare with Fig. 1) was further supported by the detection of both high molecular‐mass S‐layer glycoproteins, TfsA‐GP and TfsB‐GP, on a Coomassie Brilliant Blue‐stained SDS–PAGE gel (not shown) and on a Western immunoblot using anti‐TfsA and anti‐TfsB antibody, respectively (Fig. 2).

Considering the demonstrated involvement of the S‐layer in the immune response to *T. forsythia* (Sekot *et al*., 2011), it was important to quantify the S‐layer content of the OMVs in relation to intact *T. forsythia* cells to assess the inflammatory response triggered by the OMVs (see below). Densitometric quantification of the two S‐layer glycoproteins TfsA‐GP and TfsB‐GP from the Western immunoblot revealed that the amount of S‐layer in the OMV preparation with a total protein content of 5 μg equaled that present on 10^7^ *T. forsythia* cells.

### Proteomics

To obtain a detailed composition of *T. forsythia* ATCC 43037 OMVs, we performed a liquid chromatography‐MS/MS analysis of three independent biological replicates upon separation by SDS–PAGE, resulting in the high‐confidence identification of 208, 202 and 192 proteins, respectively. Protein hits that were exclusively detected in one (31 hits) or two replicates (23 hits) were disregarded. In all, 175 proteins were found in all three replicates and were selected for further bioinformatic analyses (Table 1). These represent 76% of all identified proteins, implying good analytical and biological reproducibility (see Supplementary material, Table S1).

We decided to use a 1D gel shotgun proteomics approach to avoid potential pitfalls of frequently used techniques based on 2D‐gel electrophoresis, where specific non‐ionic or zwitterionic detergents are required in isoelectric focusing in which highly hydrophobic membrane proteins are relatively insoluble (Braun *et al*., 2007).

#### The strain issue

Due to an error in strain attribution, all previous studies on *T. forsythia* ATCC 43037 reported in the literature are based on the genome of a different strain, *T. forsythia* 92A2 (which has only recently been deposited at ATCC under the strain number BAA‐2717). To complicate matters, most references to *T. forsythia* genes and proteins in the literature use the initial annotation that was performed by the Los Alamos National Laboratory (http://www.oralgen.org), which is different from the annotation that can be found at NCBI (http://www.ncbi.nlm.nih.gov). All our data refer to the recently sequenced strain ATCC 43037 and the new annotation for the genes (Tanf) is used.

As this is the first study to use the correct genome sequence, we searched for orthologs of the identified proteins in both the NCBI (BFO) and Oralgen (TF) annotations and included all respective locus tags in Table S1. Although some parts of the genomes of the two strains (ATCC 43037 vs. 92A2) show considerable differences (Friedrich *et al*., 2015), all but two proteins present in the OMVs have putative orthologs in strain 92A2.

#### Functional annotation

A combination of the inference of protein function via homology and a prediction using PANNZER (Koskinen *et al*., 2015) suggested functions for 97 OMV proteins, whereas the remaining 78 hits were classified as hypothetical or were only assigned general domain descriptions. When categorized according to COG functional classes (http://www.ncbi.nlm.nih.gov/COG), most proteins found in OMVs are involved in inorganic ion transport and metabolism (25 hits), post‐translational modification, protein turnover and chaperone function (12 hits), carbohydrate transport and metabolism (7), cell motility, secretion and intracellular trafficking (7), cell envelope and OM biogenesis (6) and amino acid transport and metabolism (4). The majority of proteins, a total of 106 (60%), could not be placed into any COG class (Table S1).

#### Prediction of subcellular protein localization

**Figure 3:**
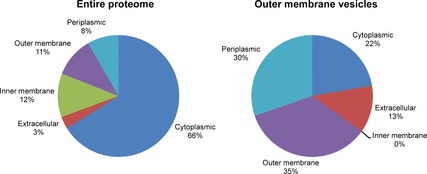
Comparison of subcellular localization prediction of the whole proteome and the outer membrane vesicle (OMV) fractions of *Tannerella forsythia* ATCC 43037.

The prediction of subcellular localization using the software CELLO places 61 proteins (35%) in the OM, 53 (30%) in the periplasm and classifies 22 proteins (13%) as extracellular proteins. When compared with the subcellular distribution within the entire *T. forsythia* proteome (Fig. 3; see Supplementary material, Table S1), OMVs are highly enriched in OM and periplasmic proteins whereas, as expected, components predicted to be associated with the inner membrane are completely excluded. Thirty‐nine proteins (22%) are predicted to be localized to the cytoplasm – however, of these, 12 have been previously identified as components of the *T. forsythia* OM proteome (Veith *et al*., 2009b), indicating the limitations of *in silico* subcellular localization prediction.

As several proteomic analyses have shown that OMVs can contain cytoplasmic proteins, either through targeted incorporation or accidental inclusion, a number of proteins predicted to originate from the cytoplasm are to be expected (Lee *et al*., 2008). Our initial OMV purifications contained two particularly abundant cytoplasmic proteins, elongation factor EF‐Tu and molecular chaperone GroEL, both of which have been frequently found in other OMV proteome studies (Ohara *et al*., 2006). Even though it is often suggested to use density gradient centrifugation or gel filtration to enhance purity, we found that harvesting the OMVs from a younger culture (here, a batch culture at OD_600_ ~1.0) and including additional washing steps eliminated the presence of these two proteins alongside any ribosomal components.

#### Glycoproteins

Based on our previous finding that *T. forsythia* modifies many of its proteins with the unique S‐layer glycan (Posch *et al*., 2011) and that *T. forsythia* manipulates the cytokine responses of macrophages and monocytes through its surface glycosylation (Settem *et al*., 2013) as well as its biofilm formation capacity (Honma *et al*., 2007), it was interesting to see whether the OMVs would contain glycosylated cargo apart from the S‐layer glycoproteins. To identify potential glycoproteins, we therefore searched the protein hits for the phylum‐wide glycosylation motif D(S,T)(A,L,V,I,M,T) (Coyne *et al*., 2013). A total of 80 proteins (46%) contain at least one occurrence of the sequon, with the S‐layer protein TfsB (Tanf_03375) and the hypothetical protein Tanf_00535 featuring the most (16 predicted sites). Although the number of identified OMV proteins comprise only 7% of the annotated proteome, they include 27% of all proteins that have a higher number of glycosylation motifs (i.e. six or more).

To the best of our knowledge, previous studies have identified a total of 16 glycoproteins in *T. forsythia*, either through the proteomic analysis of carbohydrate‐stained bands on 2D‐gels (Veith *et al*., 2009b) or the detection of the S‐layer oligosaccharide from proteins subjected to reductive β‐elimination (Posch *et al*., 2011). Using whole cell extracts of *T. forsythia*, we identified four more proteins carrying the S‐layer glycan in the course of this study (data not shown). Out of these 20 glycoproteins, we could detect 18 in the OMVs (Table 2). The glycoproteins found in the OMV fraction include, besides the two S‐layer proteins TfsA and TfsB (11 and 16 predicted glycosylation sites), and the major surface antigen BspA (one predicted glycosylation site), a possible hemagglutinin/hemolysin (Tanf_06020; six predicted glycosylation sites) and a putative zinc‐protease (Tanf_03780; two predicted glycosylation sites) that has also been found to be preferentially packed into the OMVs of *Bacteroides fragilis* (Elhenawy *et al*., 2014) and *P. gingivalis* (Veith *et al*., 2014). Also present, and presumably in the lumen of the OMVs, is a tetratricopeptide‐domain‐containing protein (Tanf_07305) that shows homology to PG1385 (TprA) from *P. gingivalis*, which is upregulated in mouse subcutaneous infection (Yoshimura *et al*., 2008). Of the 18 OMV glycoproteins, 10 are hypothetical. These include Tanf_00990, Tanf_11855, Tanf_00065, Tanf_02425 and Tanf_08965, all of which have been described as particularly abundant in the OM (Veith *et al*., 2009b).

**Table 2:** Glycoproteins identified in *Tannerella forsythia* ATCC 43037 outer membrane vesicles

ATCC locus tag	Description	MW (kDa)	Glyco sites	CTD present	References
Tanf_03375	S‐layer protein TfsB	150.4	16	+	Veith *et al*. (2009b); Posch *et al*. (2011)
Tanf_04820	surface antigen BspA	123.3	1	+	Veith *et al*. (2009b)
Tanf_03370	S‐layer protein TfsA	133.1	11	+	Veith *et al*. (2009b); Posch *et al*. (2011)
Tanf_02425	hypothetical protein	159.9	13		Veith *et al*. (2009b); Posch *et al*. (2011)
Tanf_06020	possible hemagglutinin/hemolysin	131.2	6	+	Veith *et al*. (2009b)
Tanf_08330	tetratricopeptide repeat protein	71.2	2		Veith *et al*. (2009b); Posch *et al*. (2011)
Tanf_07305	tetratricopeptide repeat protein	46.5	1		our experiments
Tanf_08965	hypothetical protein	171.4	11		Veith *et al*. (2009b); Posch *et al*. (2011)
Tanf_10985	possible lipoprotein	54.5	6		Veith *et al*. (2009b)
Tanf_04010	probable secreted glycosylhydrolase	32.6	3		Veith *et al*. (2009b)
Tanf_13170	putative peptidyl‐prolyl isomerase	61.7	4		our experiments
Tanf_13485	OM protein SusE	41.8	1		Posch *et al*. (2011)
Tanf_03780	peptidase, M16 family	107.5	2		our experiments
Tanf_00990	hypothetical protein	61.5	2		Veith *et al*. (2009b)
Tanf_00065	hypothetical protein	65.2	2		Veith *et al*. (2009b)
Tanf_11855	hypothetical protein	67.4	3		Veith *et al*. (2009b)
Tanf_09795	cationic OM protein OmpH	19.0	1		our experiments
Tanf_09955	hypothetical protein	26.1	2		Veith *et al*. (2009b)

#### CTD proteins

In *P. gingivalis*, a close phylogenetic relative of *T. forsythia* affiliated to the *Bacteroidales* order of bacteria (Conrads *et al*., 2005; Coyne *et al*., 2013) and member of the ‘red‐complex’ consortium of periodontal pathogens (Socransky *et al*., 1998), the newly identified T9SS is dependent on a CTD in certain proteins for their successful secretion and surface attachment (Glew *et al*., 2012). These CTD proteins, along with other virulence factors, were later found to be considerably enriched in the OMVs (Veith *et al*., 2014).

Two recent studies provided the first evidence of a T9SS in *T. forsythia* and simultaneously confirmed the importance of CTD signals for the secretion of the two S‐layer proteins (Narita *et al*., 2014; Tomek *et al*., 2014). To determine to which degree CTD proteins were present within the OMV preparation, we compared all putative CTD proteins from strain 92A2 (Veith *et al*., 2013) to their respective orthologs in strain ATCC 43037 using BLASTP and also directly searched for CTD‐specific motifs with GLAM2Scan. Of the 26 putative CTD proteins that we could identify in the entire *T. forsythia* ATCC 43037 proteome (see Supplementary material, Table S1), 14 were detected in the OMVs. Of these, seven were already reported to be part of the OM proteome (Veith *et al*., 2009b), while the other seven, mostly hypothetical proteins, could be exclusive to the OMV preparation. Four proteins, Tanf_00065, Tanf_00990, Tanf_08965 and Tanf_11855, show high sequence similarity to the N‐terminal region of previously reported CTD proteins (Narita *et al*., 2014) but lack the C‐terminal OM translocation signal itself.

As expected, several components of the OM‐bound T9SS could also be found in our analysis of the OMV fraction, including orthologs of PorQ (TF1959), PorT (TF0188), the putative C‐terminal signal peptidase PorU (TF0955) and LptO (TF2852). In *P. gingivalis*, the integral OM protein LptO has been shown to be essential for the coordinated secretion of CTD proteins (Chen *et al*., 2011) and was found to be among the most abundant vesicle cargo components in OMVs (Veith *et al*., 2014).

Among the protein hits with the highest Mascot score were the CTD‐containing S‐layer glycoproteins TfsA‐GP and TfsB‐GP, the surface antigen BspA and the hypothetical high‐molecular‐weight glycoprotein Tanf_02425. BspA is a member of the leucine‐rich repeat and bacterial immunoglobulin‐like protein families; it is associated with the cell surface of *T. forsythia* and functions as an important modulator of host innate immune responses through activation of TLR2 in cooperation with TLR1 (Onishi *et al*., 2008). Also, the virulence‐related possible hemagglutinin/hemolysin (Tanf_06020) is a CTD‐protein.

#### TonB‐associated OM proteins

As expected, components of the OM were the most numerous among all OMV protein hits. Ninety‐nine (57%) of all identified OMV proteins were already detected in the published OM proteome of *T. forsythia* 92A2 (Veith *et al*., 2009b).

Among them are 40 proteins that are classified as being associated with TonB‐dependent transport, 30 of which show similarity to gene products of the *susB* operon (including SusC, SusD and SusE) of *Bacteroides thetaiotaomicron*. These OM proteins form transporter complexes with TonB‐dependent receptors that are likely to be involved in the import of carbohydrates and other nutrients (Lee *et al*., 2008). One particular SusD family protein, Tanf_11260 (TF1416 in strain 92A2) has been identified as an *in vivo* antigen during human infection (Galka *et al*., 2008).

Within this group are also orthologs (Tanf_13710 and Tanf_13705) of a recently described TonB‐dependent OM sialic acid transport system in strain 92A2 consisting of the neuraminate OM permease NanO (TF0033) and the extracellular neuraminate uptake protein NanU (TF0034) (Banerji *et al*., 2008). Another component of *T. forsythia*'s sialic acid utilization locus, the sialidase NanH, could also be found in OMVs (Tanf_13700). This enzyme allows *T. forsythia* to scavenge sialic acid from human glycoconjugates, with the cleaved sialic acid acting as an important nutrient for bacterial growth as well as being key to the initial formation and maturation of biofilms (Roy *et al*., 2011).

We also identified a homolog of HmuY, a TonB‐dependent receptor‐associated OM lipoprotein that was found to be highly enriched in the vesicle membrane of *P. gingivalis* OMVs (Veith *et al*., 2014).

Interestingly, while all four aforementioned proteins could be readily detected in all three replicates of our analysis, none of them has been previously described as part of the OM proteome.

#### Hydrolytic enzymes

A previous investigation of two different *Bacteroides* species revealed that acidic glycosidases and proteases were preferentially and selectively packed into their OMVs (Elhenawy *et al*., 2014). While our analysis did not allow us to determine whether controlled enrichment of components took place, we detected a high number of proteins annotated to possess hydrolytic functions, many of which were not found previously in the OM proteome and could therefore constitute virulent OMV cargo. Apart from the sialidase NanH that was mentioned earlier, the OMV fraction contained another sialidase, SiaHI (Horstman & Kuehn, 2000) and the β‐*N*‐acetylglucosaminidase HexA (Hughes *et al*., 2003), both of which are suspected to be involved in biofilm formation (Yoshimura *et al*., 2008). The failure to detect the recently described α‐l‐fucosidase of *T. forsythia* in the bacterium's OMVs (Megson *et al*., 2015), might be explained by its biological role in the catabolism of short oligosaccharides in the periplasm, thereby only indirectly contributing to the virulence of *T. forsythia*.

In addition to orthologs of five periplasmic peptidases that have been described as constituents of the OM proteome (Veith *et al*., 2009b), we identified seven additional peptidases. Four of them, the dipeptidase Tanf_00250, the zinc protease Tanf_03780, the putative peptidase Tanf_11165 and the aminopeptidase Tanf_13550 exhibit high homology with hydrolases that have been found exclusively in the OMVs of both *B. fragilis* (Elhenawy *et al*., 2014) and *P. gingivalis* (Veith *et al*., 2014). Another periplasmic protease, Tanf_11420, is annotated as HtrA, a secreted virulence factor in *Helicobacter pylori*. HtrA has been shown to facilitate the invasion of intact epithelium by cleaving the cell‐adhesion protein E‐cadherin, thereby allowing persistent *H. pylori* colonization and pathogenesis (Hoy *et al*., 2010). A recent study identified a group of putative secretory proteases that share a nearly identical C‐terminal domain ending with a Lys‐Leu‐Ile‐Lys‐Lys motif and that are consequently referred to as KLIKK proteases (Ksiazek *et al*., 2015b). Of these, the metalloprotease forsilysin (Tanf_06225) and the serine protease miropsin‐2 (Tanf_06530) were found in the OMV proteome. Both enzymes have been shown to possess the ability to degrade elastin, an important component of connective tissue, and could therefore play an active role in periodontal lesions. In contrast to our finding of miropsin‐2 and forsilysin in the OMV fraction, these enzymes were detected as soluble forms in the particle‐free culture supernatant of *T. forsythia* cells (Narita *et al*., 2014).

Tanf_05640 is annotated as a xylanase, an enzyme that was also found in the OMVs of the cellulolytic bacterium *Fibrobacter succinogenes* (formerly in the genus *Bacteroides*), where it is believed to contribute to polymer digestion and nutrient acquisition (Forsberg *et al*., 1981). It is conceivable that the xylanase ortholog in *T. forsythia* is one of several hydrolytic enzymes involved in the scavenging of nutrients.

The lysophospholipase Tanf_10855 belongs to a family of lipolytic proteins that share a distinct GDSL motif in their active site. In the pathogen *Legionella pneumophila*, a related enzyme, lysophospholipase A, was found in the OMV fraction and could be involved in the mediation of the fusion processes between OMVs and their target membranes (Galka *et al*., 2008). Apart from their role as virulence factors in many pathogenic bacteria, phospholipases can also create cleavage products that are used in the bacteria's lipid metabolism (Banerji *et al*., 2008).

Other hydrolytic enzymes that were detected in the OMVs include a β‐galactosidase (Tanf_02010), an endonuclease (Tanf_02020), a phosphohydrolase (Tanf_13640), a putative metallopeptidase (Tanf_08225) similar to thermolysin, and an alkaline phosphatase (Tanf_00095). The periplasmic enzyme alkaline phosphatases has been reported in the OMVs of various species (Horstman & Kuehn, 2000; Bomberger *et al*., 2009; Evans *et al*., 2012) and is considered a major virulence factor in *Pseudomonas aeruginosa*.

Considering that OMVs of different species have been reported to contain a wide range of hydrolytic enzymes, it is not surprising to find adaptations to the highly proteolytic environment of subgingival plaque that *T. forsythia* inhabits. In our analyses, we detected an ortholog of miropin (Tanf_07655), a bacterial serpin that has been recently reported to inhibit a wide range of proteases and that may function as an important virulence factor by protecting the bacterium from the destructive activity of neutrophil serine proteases (Chen *et al*., 2005). While the subcellular localization prediction in our study classifies miropin as a cytoplasmic protein, Ksiazek *et al*. suggest that it is most likely exposed to the extracellular environment on the bacterial cell surface with a fraction also present in the periplasm (Ksiazek *et al*., 2015c).

#### Other proteins

Among the other integral OM proteins that could be detected in the OMV preparation is an ortholog to the major OmpA‐like protein (Tanf_10935) which has been shown to be antigenic but most likely not exposed on the surface (Veith *et al*., 2009b). Furthermore, we could find a protein that exhibits high sequence similarity to lipoprotein PG3 of *P. gingivalis* (Tanf_00475) and that has been identified as an antigen specifically expressed in patients with periodontal disease (Yoo *et al*., 2007).

Interestingly, an ortholog of the most abundant soluble protein in the lumen of *P. gingivalis* OMVs, signal protein PDZ, could also be found in our OMV analysis (Tanf_10630).

Superoxide dismutase activity predicted to be encoded by Tanf_04530 has been shown to be important in the obligate anaerobe *P. gingivalis* for protection against aerobic exposure (Ohara *et al*., 2006). The Fe/Mn‐containing superoxide dismutase SodF in *T. forsythia* has been shown to be under the regulation of a redox‐sensor which exerts an influence over both antioxidant responses and synergistic biofilm formation with *Fusobacterium nucleatum* (Honma *et al*., 2009).

#### Unclassified proteins

Despite using an automated protein function prediction method that was ranked highly in a recent, large‐scale evaluation (Radivojac *et al*., 2013), more than half of all proteins that were detected in the OMV preparation could not be assigned any function. Current protein databases are faced with exponentially growing sequencing information but a lack of experimentally verified protein annotations. Therefore, the automated annotation of proteins of uncertain function remains a challenging process (Mills *et al*., 2015).

Nevertheless, two unclassified proteins that were found in the OMVs are worth mentioning: the two tetratricopeptide repeat (TPR) domain proteins Tanf_07305 and Tanf_09615 are orthologous to proteins identified as soluble cargo in the vesicle lumen of *P. gingivalis* OMVs (Veith *et al*., 2014). One of them, the TPR‐domain protein TprA (PG1385), has recently been shown to be involved in the pathogen's virulence. The *tprA* gene was upregulated in *P. gingivalis* wild‐type cells placed in a mouse subcutaneous chamber and a *tprA* knock‐out mutant was clearly less virulent (Kondo *et al*., 2010).

### Inflammatory response in U‐937 macrophages and hPdLFs upon stimulation with OMVs

Before assaying the inflammatory response and to exclude a toxic effect of the purified OMVs on the cell lines used in this study, the viability of U937 macrophages and hPdLFs upon challenge with OMVs at concentrations of 0.03–10 μg ml^−1^ was tested (data not shown). In U937 macrophages, OMVs induced a dose‐dependent decrease in macrophage viability. This effect was significant for all tested OMV concentrations. The viability of hPdLFs was significantly increased by OMVs at concentrations ranging from 1 to 10 μg ml^−1^, whereas lower OMV concentrations had no significant effect on hPdLF viability.

**Figure 4:**
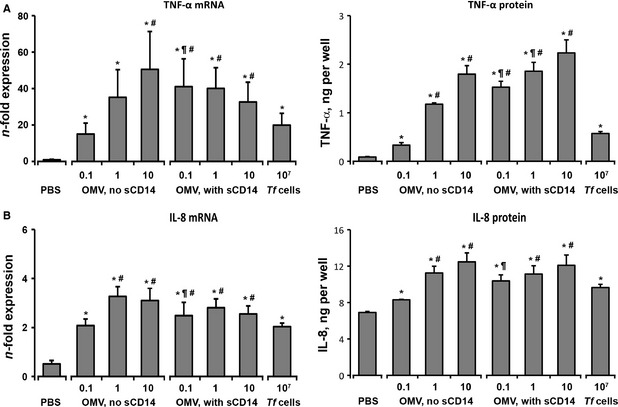
Effect of *Tannerella forsythia* ATCC 43037 outer membrane vesicles (OMVs) on the production of tumor necrosis factor‐α (TNF‐α) and interleukin‐8 (IL‐8) in U937 macrophages. Expression levels of TNF‐α (A) and IL‐8 (B) in U‐937 macrophages upon 3 h of stimulation with OMVs (0.1–10 μg ml^−1^) in the presence or absence of sCD14 (0.25 μg ml^−1^). Cells stimulated with *T. forsythia* (10^7^ cells ml^−1^) were used as positive control. Left panels show gene‐expression levels measured by quantitative polymerase chain reaction. The *y*‐axis represents the *n*‐fold expression levels of the target gene in relation to non‐stimulated cells. Right panels show the protein content in the conditioned media after stimulation measured by enzyme‐linked immunosorbent assay. Data are presented as mean ± SEM. *significantly higher vs. control; ^¶^significantly higher vs. positive control (*T. forsythia*, 10^7^ cells ml^−1^); ^#^significantly higher vs. corresponding group without sCD14.

In U‐937 macrophages, in the absence of sCD14, OMVs induced a concentration‐dependent increase in mRNA and protein levels of TNF‐α and IL‐8. The presence of sCD14 in conditioned media significantly increased TNF‐α production levels in response to 0.1–1 μg ml^−1^ OMVs and IL‐8 production in response to 0.1 μg ml^−1^ OMVs. The response of U937 to 1–10 μg ml^−1^ of OMVs was significantly higher than that to whole *T. forsythia* (10^7^ cells ml^−1^) (Fig. 4).

**Figure 5:**
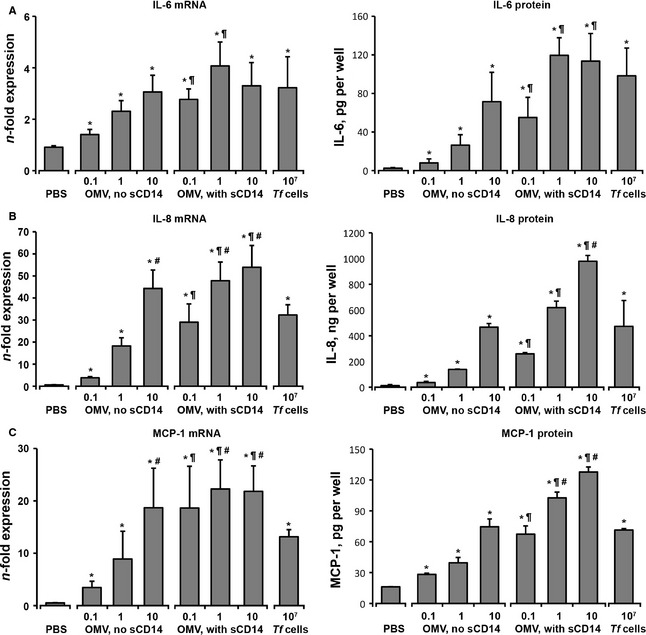
Effect of *Tannerella forsythia* ATCC 43037 outer membrane vesicles (OMVs) on the production of interleukin‐6 (IL‐6), IL‐8 and monocyte chemoattractant protein 1 (MCP‐1) by hPdLCs. Expression levels of TNF‐α (A) and IL‐8 (B) in human periodontal ligament fibroblasts (hPdLFs) upon 24 h of stimulation with OMVs (0.1–10 μg ml^−1^) in the presence or absence of sCD14 (0.25 μg ml^−1^). Cells stimulated with *T. forsythia* (10^7^ cells ml^−1^) were used as positive control. Left panels show gene‐expression levels measured by quantitative polymerase chain reaction. The *y*‐axis represents the *n*‐fold expression levels of target gene in relation to non‐stimulated cells. Right panels show protein content in the conditioned media after stimulation measured by enzyme‐linked immunosorbent assay. Data are presented as mean ± SEM. *significantly higher vs. control; ^¶^significantly higher vs. positive control (*T. forsythia*, 10^7^ cells ml^−1^); ^#^significantly higher vs. corresponding group without sCD14.

Similarly, in hPdLFs, in the absence of sCD14, OMVs induced a concentration‐dependent increase of IL‐6, IL‐8 and MCP‐1 expression, on both gene and protein levels. The presence of sCD14 significantly increased both gene and protein levels of all pro‐inflammatory mediators in response to 0.1–1 μg ml^−1^ OMV as well as protein production in response to 10 mg ml^−1^ OMV. The IL‐8 and MCP‐1 response to 10 μg ml^−1^ OMV was significantly higher than that to whole *T. forsythia* (10^7^ cells ml^−1^) (Fig. 5).

In the present study, we focused on the measurements of the expression of those pro‐inflammatory mediators that are thought to play an important role in the progression of peritonitis. TNF‐α may directly stimulate bone resorption *in vitro* and *in vivo* (Mundy, 1993); or stimulate production of prostaglandin E_2_ (Nakao *et al*., 2002; Rausch‐Fan *et al*., 2005), which is a potent stimulator of bone resorption (Offenbacher *et al*., 1993). Interleukin‐6 is a pro‐inflammatory cytokine, which plays a key role in acute inflammation phase and promotes bone resorption (Ishimi *et al*., 1990; Fonseca *et al*., 2009). Interleukin‐8 and MCP‐1 are chemoattractants that induce migration of neutrophils and monocytes, respectively, to the inflammation site and promote the development of acute inflammation (Baggiolini *et al*., 1994; Silva *et al*., 2007).

Our data showed that the response of macrophages and hPdLFs to an OMV challenge strongly depends on the presence of CD14 in the conditioned media. CD14 is a glycosylphosphatidylinositol‐anchored protein, but it also exists in soluble form (Kim *et al*., 2005). CD14 together with the LPS‐binding protein is thought to capture the LPS molecule and transfer it to TLR4 (Park *et al*., 2009). Hence, dependency of OMV response on the presence of sCD14 suggests the involvement of LPS‐signaling in this inflammatory response. The effect of sCD14 was more pronounced in hPdLF than in macrophages. This observation was not surprising, considering that macrophages are hematopoietic cells, which usually express high levels of membrane‐bound CD14 (Jersmann, 2005), whereas hPdLFs usually express low levels of membrane‐bound CD14 (Hatakeyama *et al*., 2003). It is known that soluble CD14 is present in gingival crevicular fluid and its levels might change in periodontal disease (Jin & Darveau, 2001).

## Conclusions

It is established knowledge that the periodontal pathogen *T. forsythia* has developed sophisticated strategies for host evasion and virulence, including molecular mimicry of host tissues to avoid immune detection, modulation of the host immune response, and entry into host cells to escape immune surveillance (Amano *et al*., 2014). Key to these mechanisms is, among others, the decoration of *T. forsythia's* cell surface with a unique glycan linked to its S‐layer proteins whose synthesis relies on the general protein *O*‐glycosylation pathway of the bacterium that also targets other *Tannerella* proteins (Posch *et al*., 2011, 2012).

In this study, we characterized *T. forsythia* OMVs as a new addition to the bacterium's virulence repertoire and simultaneously investigated, for the first time, OMV biogenesis of an S‐layer covered bacterium. It was shown that OMVs with a narrow size distribution of ~100 nm in diameter are formed by budding off from the OM while retaining an intact, glycosylated S‐layer (compare with Fig. 1). Hence, it is conceivable that the S‐layer serves its important functions in mediation of cell adhesion and invasion (Sakakibara *et al*., 2007) as well as in immune evasion (Sekot *et al*., 2011; Settem *et al*., 2013, 2014) also in conjunction with OMVs as a prerequisite for the delivery of virulent cargo to host cells.

Shotgun proteomics using liquid chromatography matrix assisted laser desorption/ionization time‐of‐flight/time‐of‐flight identified 175 proteins, many of which have an attributed virulence function, are (predicted) glycoproteins or both (Table 1; see Supplementary material, Table S1). Considering that OMVs selectively sort their cargo it is likely that these glycoproteins would serve important roles in OMV functionality, given the importance for virulence that has already been attributed to the *T. forsythia O*‐glycan (Settem *et al*., 2013, 2014).

Despite the clear enrichment of OM and periplasmic proteins in the *T. forsythia* OMVs alongside glycoproteins, the preparations were predicted to contain a few cytoplasmic proteins, with the numbers varying depending on the used algorithm for subcellular localization prediction. Although we consider a contamination of the OMV preparation from other subcellular compartments as unlikely, we cannot exclude at the current stage of investigation that the preparation contained small amounts of inner OM vesicles. These were recently described as a new type of membrane vesicles of Gram‐negative pathogens such as *Neisseria gonorrhoeae*,* Pseudomonas aeruginosa* and *Acinetobacter baumanii*, occurring in minor amounts (0.2–1.5% of total vesicles produced) and typically also containing cytoplasmic components (Pérez‐Cruz *et al*., 2015). On the other hand, although the number of cytoplasmic proteins found in an OMV preparation is often used as a measure of its purity, the presence of such proteins is not necessarily an indication of contamination with cellular material (Ohara *et al*., 2006).

The virulent character of *T. forsythia* OMVs was supported by the measurement of the release of the proinflammatory mediators TNF‐α and IL‐8 in macrophages and IL‐6, IL‐8 and MCP‐1 in hPdLFs. Cytokine release was dependent on the concentration of OMVs used for stimulation and, in all cases, was significantly higher than the response to a challenge with whole *T. forsythia* cells. To elucidate the mechanisms underlying these observed responses, future studies will focus on the virulence potential of particular glycosylated OMV cargo.

In conclusion, our study represents the first characterization of *T. forsythia* OMVs, their proteomic composition and immunogenic potential. Our results suggest that *T. forsythia* OMVs could play a role in the shuttling of virulence factors to affect the development and progression of periodontal disease.
